# Osthole Synergizes With HER2 Inhibitor, Trastuzumab in HER2-Overexpressed N87 Gastric Cancer by Inducing Apoptosis and Inhibition of AKT-MAPK Pathway

**DOI:** 10.3389/fphar.2018.01392

**Published:** 2018-11-27

**Authors:** Yun Yang, Feng Ren, Ziyin Tian, Wei Song, Binfeng Cheng, Zhiwei Feng

**Affiliations:** ^1^School of Basic Medical Sciences, Xinxiang Medical University, Xinxiang, China; ^2^State Key Laboratory of Antibody Medicine and Targeted Therapy, Shanghai, China; ^3^Henan Collaborative Innovation Center of Molecular Diagnosis and Laboratory Medicine, Xinxiang, China; ^4^College of Life Science and Engineering, Henan University of Urban Construction, Pingdingshan, China; ^5^School of Life Sciences and Technology, Xinxiang Medical University, Xinxiang, China

**Keywords:** trastuzumab, osthole, gastric cancer, apoptosis, AKT

## Abstract

**Background and Purpose:** Although trastuzumab has shown considerable activity in the treatment of HER2-positive breast and gastric cancers, a significant proportion of patients do not respond to trastuzumab. Recent studies revealed that osthole, an active coumarin isolated from *Cnidium monnieri* (L.) Cusson possesses potent anti-tumor activity. Here, we for the first time investigated the anti-tumor activity of trastuzumab in combination with osthole in HER2-overexpressing cancers.

**Materials and Methods:** N87 and SK-BR-3 cell lines, which were HER2-overexpressing cancer cells were used in our study. Cell Counting Kit-8 (CCK-8) assay was utilized to test the inhibitory effects of trastuzumab plus osthole. Combination index (CI) values were calculated using the Chou-Talalay method. Fluorescence-Activated Cell Sorter (FACS) assay was used to examine the cell cycle change and apoptosis upon combinatorial treatment. N87 tumor xenografts were established to evaluate *in vivo* effects of trastuzumab plus osthole. In addition, molecular mechanisms were analyzed by Western blot *in vitro and in vivo*.

**Results:** As shown in our study, osthole alone exhibited effective anti-tumor activity against HER2-overexpressed N87 gastric cancer cells and SK-BR-3 breast cancer cells, which may be attributed to cell cycle arrest on G2/M phase and apoptosis. More importantly, our data demonstrated that trastuzumab plus osthole was much more potent than either agent alone in inhibiting the growth of N87 cancer cells *in vitro* and *in vivo*, which may be partly explained by the enhanced apoptosis upon the combinatorial treatment. Besides these, we also observed a significant decrease on the phosphorylation of AKT and MAPK in N87 cells when treated with trastuzumab plus osthole compared to either agent alone. Further data from N87 tumor xenografts revealed that trastuzumab plus osthole exerted their synergistic effects mainly on AKT signaling pathway.

**Conclusion:** Collectively, these results support the clinical development of combination osthole with trastuzumab for the treatment of HER2-overexpressed gastric cancer.

## Introduction

Amplification of human epidermal growth factor receptor-2 (HER2), an important member of the ErbB family, is found in many solid tumors such as breast cancer and gastric cancer (Han et al., 2014; Yang et al., 2017). HER2 activation is dependent on HER2 homodimers or heterodimers with other ErbB family members, which could stimulate constitutive phosphorylation of HER2 and initiate the key downstream PI3K/AKT pathway or MAPK pathway that results in tumor growth and progression (Agus et al., 2002; Baselga and Swain, 2009; Wang et al., 2017). Trastuzumab is a well-known HER2-targeted humanized antibody that binds to the extracellular domain IV of HER2 and then causes inhibition of activation of downstream pathway (Wang et al., 2012; Li et al., 2013). It was approved by the US Food and Drug Administration (FDA) for clinical use for patients with HER2-overexpressing metastatic breast cancer in 1998, and for HER2-positive metastatic gastric cancer in 2010 (Baselga and Swain, 2009; Zheng et al., 2014). Despite the effectiveness, the majority of trastuzumab-responsive patients developed resistance within 1 year of treatment (Han et al., 2014; Zheng et al., 2014; Yang et al., 2017). Increased levels of membrane-bound EGFR and HER3 or sustained PI3K-AKT pathway activation has been implicated in the resistance to trastuzumab (Baselga and Swain, 2009). Collectively, there is an urgent need to enhance the efficacy of trastuzumab therapy.

Osthole is a natural coumarin, which was first derived from *Cnidium monnieri* (L.) Cusson (Zhang et al., 2015). As we know, osthole has been used in Traditional Chinese Medicine (TCM) for the treatment of cutaneous pruritus, eczema, trichomonas vaginalis infection, and sexual dysfunction for a long time (You et al., 2009; Zhang et al., 2012). Studies also revealed that osthole exhibited many pharmacological and biological activities, including anti-oxidation, anti-osteoporosis, and anti-inflammation (Liao et al., 2010; Chen et al., 2011). Recently, osthole was found to potently inhibit the growth of several types of cancer (Yang et al., 2003; Ye et al., 2013; Wang et al., 2015). However, its molecular mechanism has not been comprehensively elucidated although osthole has shown potent anti-tumor effects. Xu et al. (2011) revealed that osthole treatment caused G2/M arrest and apoptosis via modulating PI3K/Akt signaling pathway in lung cancer A549 cells. Besides, osthole was found to inhibit invasion and metastasis through down-regulation of MMP-5 and MMP-9 level in human lung adenocarcinoma cells (Kao et al., 2012). Moreover, studies revealed that osthole exerted anti-tumor effects on HER2-overexpressed breast cancer through inhibiting the c-Met/Akt/mTOR pathway (Lin et al., 2010; Hung et al., 2011). However, the anti-tumor activity of trastuzumab plus osthole in HER2-overexpressed cancers has not yet been reported.

Herein, we first investigated the anti-tumor effects of osthole alone in HER2-overexpressed N87 gastric cancer cells and SK-BR-3 breast cancer cells. Results revealed that osthole caused G2/M arrest and apoptosis in the two types of cancer cells, especially in SK-BR-3 cells. As we know, trastuzumab was an established anti-tumor therapeutic in treating HER2-positive breast cancer and gastric cancer (Baselga and Swain, 2009; Zheng et al., 2014). Next, we examined the anti-tumor activity of trastuzumab in combination with osthole against N87 and SK-BR-3 cells. Surprisingly, our results for the first time showed that osthole synergistically enhanced the growth-inhibitory effect of trastuzumab against N87 cancer cells *in vitro* and *in vivo*. Moreover, we found that the combination was more potent in inducing apoptosis and reducing the phosphorylation of AKT and MAPK than either agent alone in N87 cells, which may explain the synergistic effect. To conclude, these results shown in our study suggested that the effective regimen by combing trastuzumab with osthole has a great potential to treat HER2-overexpressed gastric cancer in clinics.

## Materials and Methods

### Cell Lines

The human breast cancer cell line SK-BR-3 and gastric cancer cell line N87 were purchased from the American Type Culture Collection (ATCC).

### Agents

Osthole was purchased from Shanghai Macklin Biochemical Co., Ltd. (Shanghai, China). It is over 99% pure determined by HPLC. The stock solution of osthole was prepared by dissolving in DMEM with 0.25% ethanol and 0.25% dimethyl sulfoxide (DMSO).

### Animals

All experimental protocols were approved by the Animal Experimentation Ethics Committee of Xinxiang Medical University and all efforts were made to minimize animal suffering and reduce the number of animals used. All experiments were performed in accordance with the guideline of the Animal Care and Use Committee of Xinxiang Medical University. Five-week-old female BALB/c nude mice were obtained from the Beijing Vital River Laboratory Animal Technology Co., Ltd. (Beijing, China).

### *In vitro* Cytotoxicity Assays

Cells were plated at a density of 5 × 10^3^ per well and incubated with increasing concentrations of osthole, trastuzumab or the combination. Two days later, cell proliferation was determined using CCK-8 kit (Dojindo, Japan). The percentage of surviving cells was calculated using the following formula: [(A450 of experiment – A450 of background)/(A450 of untreated control – A450 of background)] × 100. Combination index (CI) values were calculated using the Chou-Talalay method by Compusyn software (Han et al., 2014). Drug synergy, addition, and antagonism are defined by C.I. values less than 1.0, equal to 1.0, or greater than 1.0, respectively.

### *In vivo* Therapy Study

N87 cells (1 × 10^7^ per mouse) were inoculated subcutaneously into the right flank of female BALB/c nude mice. When tumor volumes reached an average of about 150 mm^3^ on day 8 after inoculation, the mice were randomly divided into four groups of six mice each. Mice were intraperitoneally injected with control IgG (15 mg/kg for two times every week), trastuzumab (15 mg/kg for two times every week), osthole (100 mg/kg once daily) or the combination of trastuzumab (15 mg/kg for two times every week), and osthole (100 mg/kg once daily) for 2 weeks. Tumors were measured with digital calipers, and tumor volumes were calculated by the formula: Volume = Length × (Width)^2^/2.

### Immunoblotting

Western blot was performed using established procedures.(Yang et al., 2017) Cells were lysed in lysis buffer (Beijing Dingguo Biotechnology Co., Ltd.), incubated on ice for 30 min and centrifuged for 20 min to remove cell debris. Total cell lysates were subjected to SDS–polyacrylamide and immunoblotted with primary antibodies and HRP-conjugated secondary antibody. After another wash of the membrane, the bands were detected using a super-sensitive ECL solution (Boster Biological Technology Co., Ltd., China), and visualized using an Amersham imager 600 (GE Healthcare Life Sciences, Fairfield, CT, United States).

### Cell Cycle Analysis

This assay was performed according to previous report.(Kovtun et al., 2010) Cells (1 × 10^5^/mL) were incubated with osthole for 0, 6, or 12 h at 37° C. Cells were then fixed with 1 mL of 70% ethanol, and DNA content was determined after staining with propidium iodide by flow cytometry. Flow cytometric data were analyzed using FlowJo 7.6 software.

### Apoptosis Analysis

Apoptosis analysis was performed by flow cytometry using established procedures (Zhang et al., 2012). For flow cytometry analysis, N87 cells (5 × 10^6^/well) were plated in 6-well plate and treated with osthole (40 μM), trastuzumab (10 μg/mL), or osthole (40 μM) in combination with trastuzumab (10 μg/mL) for 30 h at 37°C. The cells were then labeled with Annexin V and Propidium Iodide (PI; Beijing Dingguo Biotechnology Co., Ltd, Beijing). Apoptotic rates were determined by FACSCalibur flow cytometer (BD Biosciences, Franklin Lakes, NJ, United States) and analyzed by Flowjo software. The percentage of the early apoptosis was calculated by Annexin V (+) and PI (−), while the percentage of the late apoptosis was calculated by Annexin V (+) and PI (+).

### Statistical Analysis

Statistical analysis was performed by Student’s unpaired *t* test to identify significant differences unless otherwise indicated. Differences were considered significant at *p* < 0.05.

## Results

### Osthole Exhibits Growth-Inhibitory Activity Against HER2-Overexpressed N87 and SK-BR-3 Cancer Cells Through Cell Cycle Arrest and Apoptosis

**FIGURE 1:**
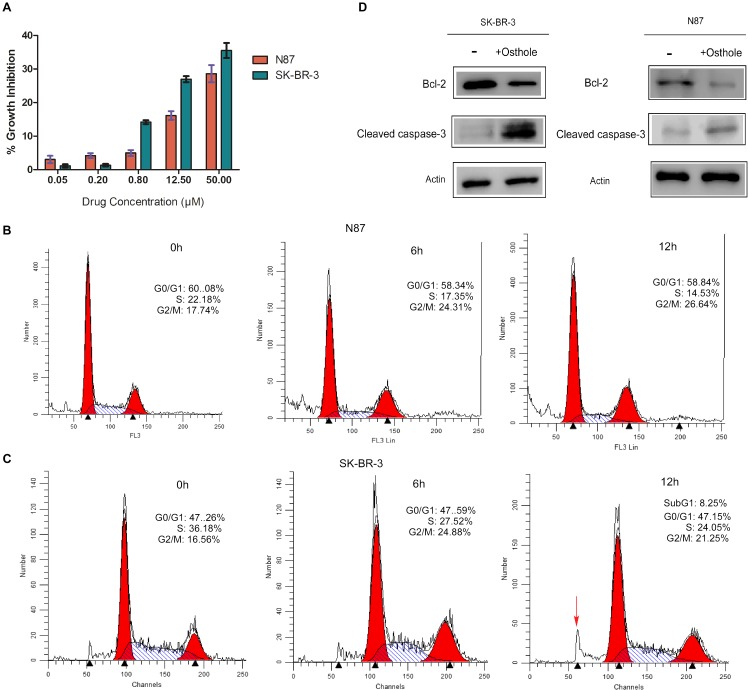
Osthole inhibited the growth of N87 and SK-BR-3 cells and induced cell cycle arrest and apoptosis. **(A)** CCK-8 assay evaluating cell growth of N87 and SK-BR-3 cells upon treatment with increasing concentration of osthole for 48 h. **(B)** Cell cycle analysis of N87 cells following 40 μM osthole treatment for 0, 6, and 12 h by flow cytometry. **(C)** Effects of osthole on cell cycle of SK-BR-3 cells. **(D)** N87 and SK-BR-3 cells were treated with 40 μM osthole for 30 h and cleaved Caspase-3 and Bcl-2 were examined by Western blot.

We first examined the inhibitory effects of osthole alone on N87 and SK-BR-3 cell lines. As shown in Figure 1A, osthole inhibited the growth of N87 and SK-BR-3 cancer cells in a dose-dependent manner. Additionally, we found that SK-BR-3 cell line responded more sensitively to osthole compared with N87 cell line.

Furthermore, we investigated the effect of osthole on cell cycle arrest and apoptosis in N87 and SK-BR-3 cells. FACS assay showing that osthole significantly elevated the percentage of G2/M phase in both N87 and SK-BR-3 cells when treated for 6 and 12 h compared to control (Figures 1B,C). More importantly, elevated sub-G1 population in SK-BR-3 cells was observed after treatment for 12 h. As we know, Bcl-2 was an important anti-apoptotic protein that regulates a late step in the apoptosis pathway (Srinivas et al., 2000; Willis et al., 2007). And Caspase-3 is an important member in Caspase family, which is critical for cytochrome c-dependent apoptosis (Zou et al., 1997). In our study, we found that Bcl-2 was down-regulated and cleaved Caspase-3 was up-regulated after treatment with osthole for 30 h, suggesting apoptosis may be induced following cell cycle arrest in response to osthole treatment in SK-BR-3 and N87 cells (Figure 1D and Supplementary Figure S1). Taken together, osthole may exert its anti-tumor effects in SK-BR-3 and N87 cells through inducing cell cycle arrest and apoptosis.

### Trastuzumab and Osthole Act Synergistically on N87 Gastric Cancer Cells *in vitro*

**FIGURE 2:**
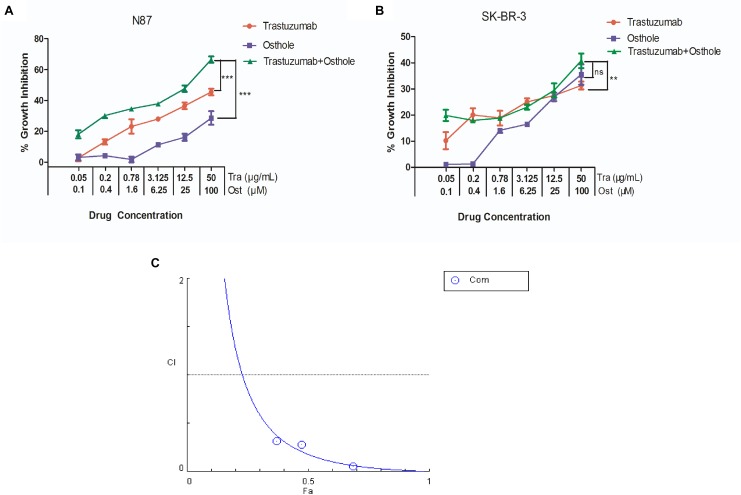
Osthole and trastuzumab synergistically inhibited the *in vitro* growth of N87 cells. **(A)** The inhibitory effects of osthole and trastuzumab combinatorial treatment against N87 cells for 48 h. **(B)** The inhibitory effects of osthole and trastuzumab combinatorial treatment against SK-BR-3 cells for 48 h. **(C)** The synergistic effect of trastuzumab in combination with osthole on the growth of N87 cell line. Combination index (CI) values were calculated at the drug concentration of trastuzumab (3.125 μg/mL) plus osthole (6.25 μM), trastuzumab (12.5 μg/mL) plus osthole (25 μM), trastuzumab (50 μg/mL) plus osthole (100 μM) using the Chou-Talalay method. Drug synergy, addition, and antagonism are defined by C.I. values less than 1.0, equal to 1.0, or greater than 1.0, respectively. Data show the mean ± SD (three independent experiments); ^∗^*p* < 0.05; ^∗∗^*p* < 0.01; ^∗∗∗^*p* < 0.001. ns, no significant difference.

Next, we examined the inhibitory effects of trastuzumab in combination with osthole on N87 and SK-BR-3 cell lines. As shown in Figures 2A,B, trastuzumab plus osthole exhibited a significantly greater inhibitory activity than either agent alone in N87 cells, while no marked synergistic effect was found in SK-BR-3 cells.

To further examine whether the combination of trastuzumab with osthole is synergistic, we treated N87 cells with combination of trastuzumab and osthole at various concentrations. Data were analyzed using the method of Chou and Talalay to establish drug C.I. values (Han et al., 2014). Synergy is defined as C.I. values of <1.0, antagonism as C.I. values >1.0, and additivity as CI values equal to 1.0. Our results showed that trastuzumab and osthole synergistically inhibited the growth of N87 cells (Figure 2C).

### Trastuzumab in Combination With Osthole Synergistically Induced Apoptosis

**FIGURE 3:**
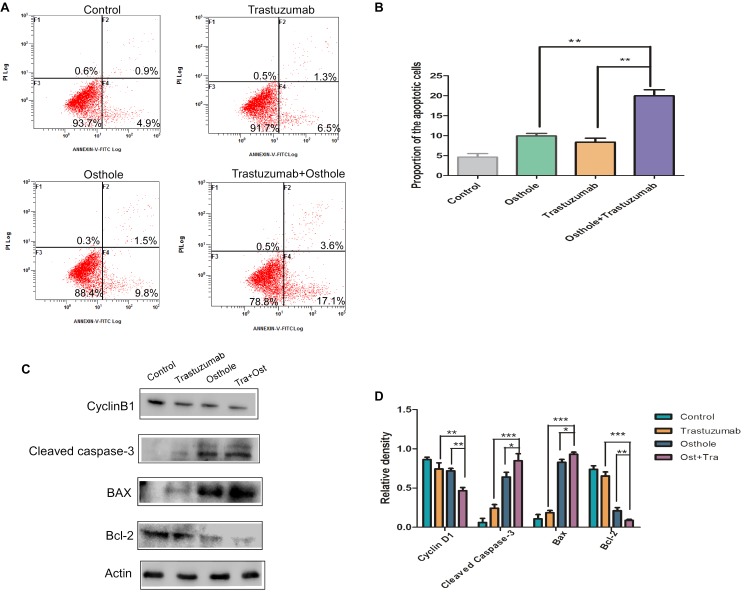
Trastuzumab enhanced osthole-induced apoptosis, which may partly explained the synergistic anti-tumor effect of trastuzumab in combination with osthole. **(A)** Induction of apoptosis of N87 cells after control IgG (10 μg/mL), osthole (40 μM), trastuzumab (10 μg/mL) or the combinatorial treatment for 30 h. Apoptosis was measured by flow cytometry. **(B)** Statistical analysis of the percentages of the apoptotic cells. Data was shown with mean ± SD. **(C)** Cell cycle and apoptosis related protein (CyclinB1, cleaved Caspase-3, Bax or Bcl-2) was examined in N87 cells when treated with control IgG (10 μg/mL), osthole (40 μM), trastuzumab (10 μg/mL), or the combination for 30 h. **(D)** Quantification of Western blot signal intensity analysis is expressed relative to the β-actin loading control by using Image J software. Data show the mean ± SD (three independent experiments); ^∗^*p* < 0.05; ^∗∗^*p* < 0.01; ^∗∗∗^*p* < 0.001.

Furthermore, we investigated whether the co-treatment of trastuzumab with osthole may synergistically induce apoptosis in N87 cells. First, the apoptotic cell percentage was analyzed by flow cytometry following Annexin V and PI staining. Results showed that the percentage of apoptotic cells was significantly increased in the trastuzumab plus osthole treated cells compared to either agent mono-treated cells (Figures 3A,B).

And we further assessed the cell extracts for expression of apoptotic markers including cleaved Caspase-3, Bcl-2, and Bax. Compared to treatment with either agent alone, combinatorial treatment significantly up-regulated the level of cleaved Caspase-3 (Figures 3C,D). In addition, Bcl-2 was markedly down-regulated, while Bax that was a protein favoring induction of apoptosis was up-regulated in trastuzumab plus osthole treated cells. Besides these, the expression of cell cycle-related molecule, CyclinB1 was significantly decreased in N87 cells upon combinatorial treatment. Taken together, these results suggested the addition of trastuzumab markedly enhanced osthole-induced apoptosis, which may partly explain the superiority of combinatorial treatment.

### Effect of Trastuzumab Plus Osthole on AKT and MAPK Signaling Pathway

**FIGURE 4:**
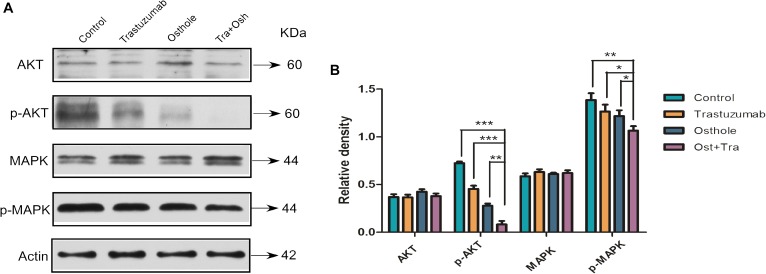
Trastuzumab in combination with osthole blocked AKT pathway in a synergistic manner. **(A)** Immunoblots assessing AKT and MAPK signaling in the N87 cell lines upon treatment with control IgG (10 μg/mL), trastuzumab (10 μg/mL), osthole (40 μM), or trastuzumab (10 μg/mL) plus osthole (40 μM) for 30 h. Data are representative of three independent experiments. **(B)** Quantification of Western blot signal intensity analysis is expressed relative to the β-actin loading control by using Image J software. Data show the mean ± SD (three independent experiments); ^∗^*p* < 0.05; ^∗∗^*p* < 0.01; ^∗∗∗^*p* < 0.001.

To further investigate the mechanism that may explain the synergistic effect, we examined the level of AKT, phosphorylated AKT, MAPK, and phosphorylated MAPK in N87 cells treated with trastuzumab in combination with osthole. Compared to trastuzumab or osthole treatment alone, trastuzumab plus osthole more significantly inhibited the phosphorylation of both AKT and MAPK in N87 cell lines (Figures 4A,B). Notably, combinatorial treatment resulted in a more effective inhibition on phospho-AKT level than on phospho-MAPK level, whereas there was no substantially decrease in total AKT and MAPK protein levels. Therefore, our results suggested that trastuzumab in combination with osthole may exert their synergistic effect on inhibiting AKT and MAPK pathway, mainly inhibiting the phosphorylation of AKT, which also further explained the superior effects of trastuzumab plus osthole.

### Trastuzumab in Combination With Osthole Potently Suppresses the *in vivo* Growth of N87 Cancer Xenografts

**FIGURE 5:**
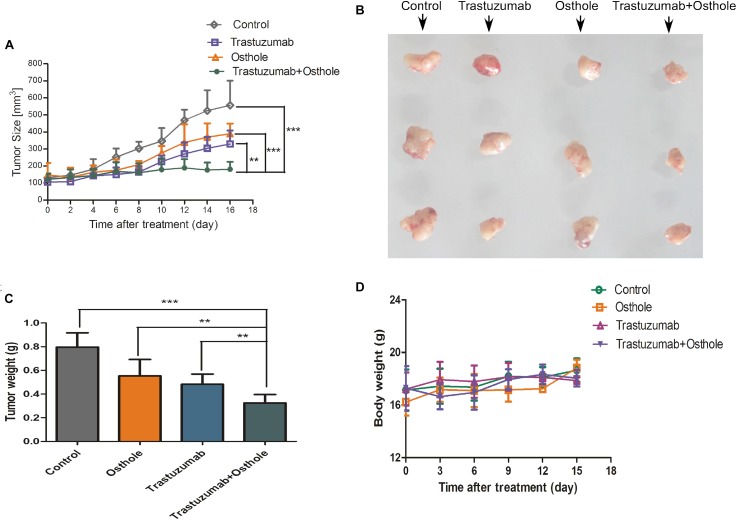
Trastuzumab plus osthole combinatorial treatment inhibits the growth of N87 cancer cells *in vivo*. **(A)** Tumor volume of N87 xenografts after injection with control IgG (15 mg/kg), Trastuzumab (15 mg/kg), Osthole (100 mg/kg), or Trastuzumab (15 mg/kg) plus Osthole (100 mg/kg). **(B)** On day 16 post first injection, xenograft tumors from each group were removed and photographed. Representative tumors in each group were shown. **(C)** After xenograft tumors were removed, these tumors were weighted. **(D)** Effects of agents on tumor-bearing mice body weight were determined using N87 tumor-bearing nude mice. Mice were weighed at regular intervals during the whole period to monitor unspecific toxicity. Data are shown as mean ± SD. (*n* = 6 mice, each group); ^∗∗^*p* < 0.01; ^∗∗∗^*p* < 0.001.

To assess the synergistic effect *in vivo*, we examined the therapeutic efficacy of trastuzumab plus osthole for nude mice bearing established N87 tumor xenografts. As shown in Figures 5A,B and Supplementary Figure S2, our *in vivo* experiments showed that the combinatorial therapy of trastuzumab with osthole significantly reduced tumor growth compared to either agent treatment alone. Compared to the control IgG, the treatment with trastuzumab and osthole combination resulted in a 50 % reduction in tumor weight (Figure 5C). Consistent with the observations *in vitro*, combinatorial treatment of trastuzumab with osthole resulted in a significant benefit over either agent alone in the N87 xenograft model. Moreover, we also preliminarily evaluated the unspecific-toxicity in these xenografts. As shown in Figure 5D, No marked weight loss was observed in trastuzumab plus osthole treated mice compared with that of in the control IgG treated group (*p* = 0.1934). Thus, our results showed that trastuzumab in combination with osthole exhibited potent inhibitory effects and good tolerance on N87 tumor xenografts.

### Trastuzumab in Combination With Osthole Inhibited AKT Signaling Pathway *in vivo*

**FIGURE 6:**
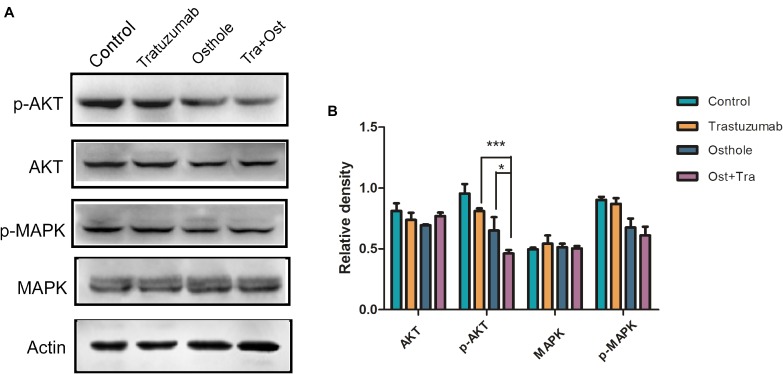
Trastuzumab in combination with osthole inhibited AKT signaling pathway *in vivo*. **(A)** Tumor tissues isolated from N87 xenografts upon treatment with control IgG (15 mg/kg), Trastuzumab (15 mg/kg), Osthole (100 mg/kg), Sor Trastuzumab (15 mg/kg) plus Osthole (100 mg/kg) were then subjected to Western blot to detect the expression of AKT, p-AKT, MAPK and p-AMPK. Data are representative of three independent experiments. **(B)** Quantification of Western blot signal intensity analysis is expressed relative to the -actin loading control by using Image J software. Data show the mean ± SD (three independent experiments); ^∗^*p* < 0.05; ^∗∗^*p* < 0.01; ^∗∗∗^*p* < 0.001.

To further determine if combinatorial treatment caused inhibition of intracellular signaling cascade *in vivo*, we examined tumor samples from treated animals using western blot assay to evaluate the degree to which MAPK or AKT signaling was inhibited. As expected, the level of pAKT in tumors of combinatorial treatment group was more effectively regressed compared to that of in trastuzumab or osthole treatment group while the level of pMAPK was not substantially reduced in tumors from trastuzumab plus osthole treated mice (Figures 6A,B). Collectively, these results above may also suggest that trastuzumab plus osthole exerted their synergistic effects mainly on AKT signaling pathway in N87 tumor xenografts.

## Discussion

In our study, we for the first time reported the anti-tumor effects of trastuzumab in combination with osthole, a natural coumarin derivative extracted from Traditional Chinese Medicine on N87 gastric cancer cells and investigated the underlying mechanism involved. We first examined the inhibitory effects of osthole on HER2-amplified N87 and SK-BR-3 cells. Results revealed that osthole exhibited potent anti-tumor activity on the two cell lines, especially on SK-BR-3 cells. Previous studies suggested that osthole could induce G2/M arrest and apoptosis in lung cancer A549 cells and hepatocellular carcinoma HepG2 cells (Xu et al., 2011; Chao et al., 2014). In consistent with these studies, we also found that osthole induced G2/M arrest and apoptosis in HER2-amplified N87 and SK-BR-3 cells. As we know, trastuzumab is a FDA-approved antibody therapeutic that has shown clinical efficacy in treating breast and gastric cancers (Hudis, 2007; Rose and Bekaii-Saab, 2011). Despite the effectiveness, numbers of patients with HER2-positive cancer treated with trastuzumab monotherapy exhibited *de novo* resistance unfortunately (Zhang et al., 2011). Thus, novel therapeutic regimens are urgently needed to enhance the efficacy of trastuzumab-therapy. Surprisingly, we found that osthole could synergistically enhance the inhibitory effect of trastuzumab against HER2-overexpressed N87 cells both *in vitro* and *in vivo*. However, the synergistic effect were not been observed in SK-BR-3 cells, which was the other trastuzumab-sensitive breast cancer cell line. The underlying mechanism explaining the different responses to trastuzumab plus osthole in the two HER2-overexpressed cancer cell lines will be further explored in our following research.

As previously reported, trastuzumab may exert its anti-tumor activity on HER2-overexpressed cancers through inducing apoptosis (Cuello et al., 2001; Milella et al., 2004). And osthole also caused cell cycle arrest and apoptosis in several types of cancer (Xu et al., 2011; Chao et al., 2014; Wang et al., 2016). In our present study, the hypothesis was investigated that if trastuzumab plus osthole may synergistically enhance the effect of apoptosis in N87 cells. As expectedly, our data revealed that trastuzumab in combination with osthole more effectively promoted apoptosis compared to either agent treatment alone.

As we know, studies have demonstrated that PI3K-AKT pathway activity is directly linked to the proliferation and growth of HER2-overexpressing cancer cells and trastuzumab mainly exerted its anti-tumor in inhibiting the HER2-PI3K-AKT pathway (Pal and Mandal, 2012; Li et al., 2013; Han et al., 2014). Recently, Lin et al. (2014) indicated that osthole inhibited IGF-1-induced EMT by blocking PI3K-Akt pathway in brain cancer cells. In our study, we also observed AKT and MAPK phosphorylation were regressed in N87 cells when treated with trastuzumab plus osthole. Especially, AKT phosphorylation was more markedly inhibited in the combinatorial treatment compared to either agent treatment alone, which was also verified in tumor samples from N87 tumor xenografts. Generally speaking, our study partly explained the molecular mechanism involved in the synergistic effects of trastuzumab in combination with osthole on HER2-overexpressed gastric cancer, which may provide a reference for other researchers. In our following study, we will explore if other AKT involved signaling pathway like c-Met/Akt/mTOR pathway may be related to the synergistic anti-tumor effects.

Taken together, our results suggested that osthole, a promising lead compound from traditional Chinese medicine could effectively inhibit N87 and SK-BR-3 cells with HER2-overexpression by causing cell cycle arrest and inducing apoptosis. More importantly, we found that combination of trastuzumab with osthole showed synergistic inhibitory effects on the growth of N87 cells, which may be partly attributed to the enhanced apoptosis. Phosphorylation of AKT were effectively inhibited *in vitro* and *in vivo* when treated with trastuzumab plus osthole may also contribute to the synergistic effect. Therefore, combination of trastuzumab with osthole provides a new strategy for targeting HER2-overexpressed gastric cancer, which will contribute to enhancing the therapeutic effect of trastuzumab. Based on these results, our study also suggested that osthole can be developed into an adjuvant drug for HER2-targeted therapy in treating HER2-overexpressed gastric cancer. In addition, a novel antibody-drug conjugate may also be designed by conjugating osthole to trastuzumab, which may represent a new therapeutic approach.

## Conclusion

Our results indicated that osthole alone exhibited effective anti-tumor activity against HER2-overexpressed N87 gastric cancer cells and SK-BR-3 breast cancer cells. Furthermore, osthole could synergistically enhance the inhibitory effect of trastuzumab against HER2-overexpressed N87 cells both *in vitro* and *in vivo*. Moreover, we explored the molecular mechanism involved in the synergistic effects, which may be attributed to the enhanced apoptosis effects and AKT-MAPK signaling pathway blockade. Collectively, these results support the clinical development of osthole plus trastuzumab for the treatment of HER2-overexpressed gastric cancer. Besides, our study may also provide a strategy for testing combinations of HER2-targeting agents with other bioactive constituents isolated from food in clinical studies.

## Data Availability Statement

Data arising from this study are contained within the manuscript.

## Ethics Statement

This study was carried out in accordance with the Principle of Laboratory Animal Care (NIH Publication No. 85-23, revised 1985). The protocol was approved by the Animal Ethics Committee of Xinxiang Medical University.

## Author Contributions

YY and ZF designed the experiments and wrote the manuscript. YY, FR, ZT, WS, and BC carried out the experiments. ZF supervised and corrected the manuscript.

## Conflict of Interest Statement

The authors declare that the research was conducted in the absence of any commercial or financial relationships that could be construed as a potential conflict of interest.
