# Supplementary material for: Osthole Synergizes With HER2 Inhibitor, Trastuzumab in HER2-Overexpressed N87 Gastric Cancer by Inducing Apoptosis and Inhibition of AKT-MAPK Pathway
Source: Front Pharmacol. 2018 Nov 27;9:1392. doi: 10.3389/fphar.2018.01392 (PMC6277458; doi:10.3389/fphar.2018.01392)
Supplement: Supplementary file 1 [file Table_1.DOCX]

Supplementary Material

Osthole synergizes with HER2 inhibitor, trastuzumab in HER2-overexpressed N87 gastric cancer by inducing apoptosis and inhibition of AKT-MAPK pathway

Yun Yang^1, 2, 5,*^, Feng Ren^1^, Ziyin Tian^1^, Wei Song^3^, Binfeng Cheng^4^, and Zhiwei Feng^1,*^

1 School of Basic Medical Sciences, Xinxiang Medical University, Xinxiang, China;

2 State Key Laboratory of Antibody Medicine and Targeted Therapy, Shanghai, China;

3 College of Life Science and Engineering, Henan University of Urban Construction, Pingdingshan, China;

4 School of Life Sciences and Technology, Xinxiang Medical University, Xinxiang, China;

5 Henan Collaborative Innovation Center of Molecular Diagnosis and Laboratory Medicine, Xinxiang, China;

* Correspondence: Yun Yang and Zhiwei Feng

E-mail address: jamesyangyun1@126.com and 123066@xxmu.edu.cn


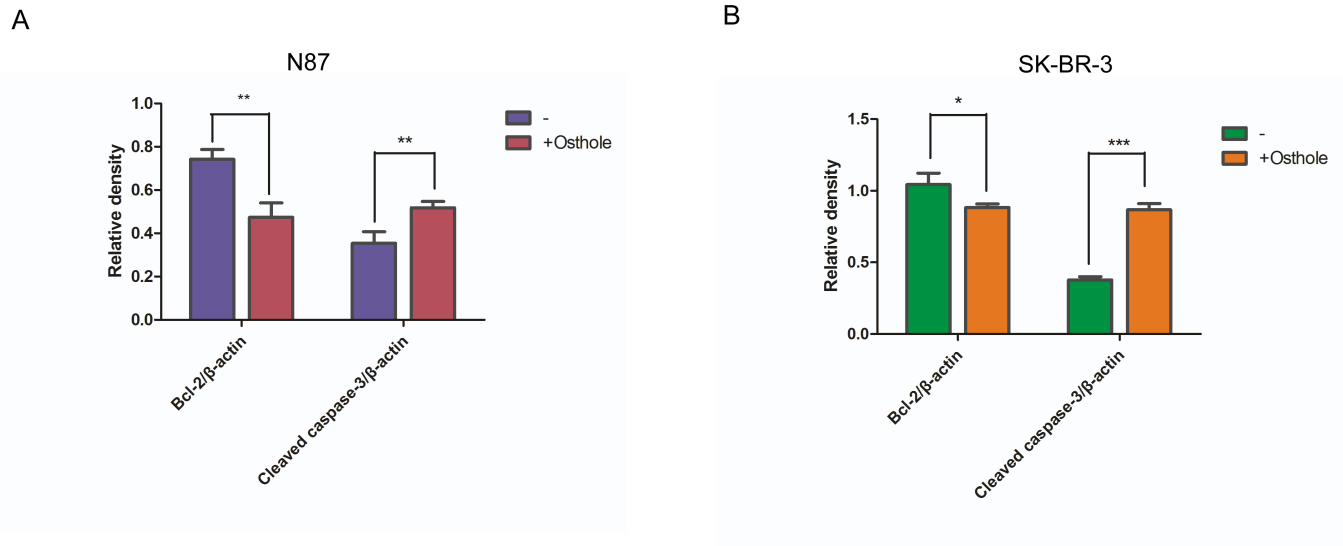


**Supplementary Figure 1.** **Quantification of Western blot signal intensity analysis in N87 and SK-BR-3 cells upon osthole treatment.** (A) N87 cells were treated with 40 μM osthole and then Cleaved caspase-3 and Bcl-2 were examined by Western blot. Quantification of Western blot signal intensity analysis is expressed relative to the β-actin loading control by using Image J software. (B) SK-BR-3 cells were treated with 40 μM osthole and then Cleaved caspase-3 and Bcl-2 were examined by Western blot. Quantification of Western blot signal intensity analysis is expressed relative to the β-actin loading control by using Image J software. Data show the mean ± SD (3 independent experiments); *, p < 0.05; **, p < 0.01; ***, p < 0.001.

**
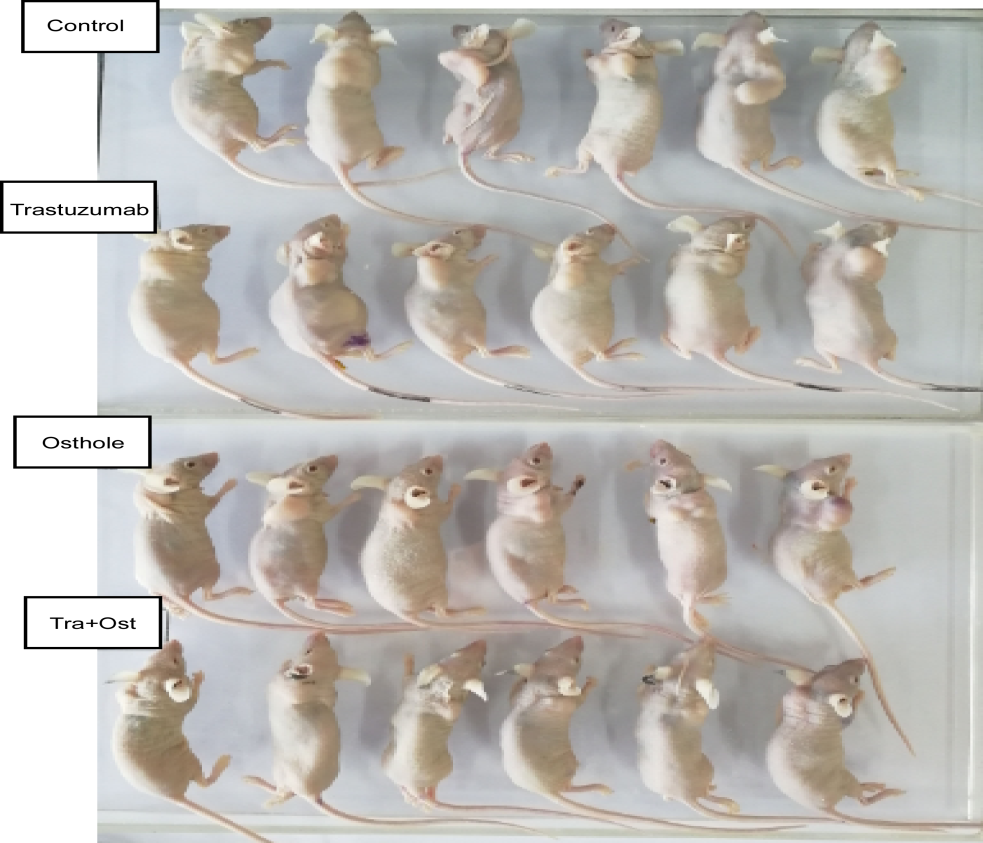
**

**Supplementary Figure 2. Anti-tumor effects of trastuzumab plus osthole on N87 tumor-bearing mice.** N87tumor-bearing mice were treated with control IgG, trastuzumab, osthole or trastuzumab plus osthole and these mice bearing tumors were photographed on day 16 post first injection.
